# Evaluation of Adrenal Function in Nonhospitalized Patients with Cirrhosis

**DOI:** 10.1155/2017/2354253

**Published:** 2017-07-24

**Authors:** Maryam Moini, Mitra Yazdani Sarvestani, Mesbah Shams, Masood Nomovi

**Affiliations:** ^1^Gastroenterohepatology Research Center, Shiraz University of Medical Sciences, Nemazee Hospital, Zand Street, Shiraz 71935-1311, Iran; ^2^Department of Internal Medicine, Fasa University of Medical Sciences, Ebne Sina Square, Fasa, Iran; ^3^Endocrinology and Metabolism Research Center, Shiraz University of Medical Sciences, Shiraz, Iran; ^4^Department of Internal Medicine, Shiraz University of Medical Sciences, Shiraz, Iran

## Abstract

**Background:**

Patients with cirrhosis and advancing hepatic insufficiency may show various degrees of other organ malfunction, including brain, kidney, and lung. Several studies have also shown a high prevalence of adrenal insufficiency in cirrhotic patients that may cause hemodynamic instability.

**Materials and Methods:**

In this study we prospectively evaluated adrenal function in a population of nonhospitalized cirrhotic patients. Categorization of liver disease severity was done according to model for end-stage liver disease (MELD) score. Adrenocorticotropic hormone stimulation testing was performed on subjects using 250 *μ*g of synthetic short acting hormone; radio immunoassay was used to measure plasma cortisol levels.

**Results:**

Of 105 cirrhotic patients, 15.23% had evidence of adrenal insufficiency. These patients were not statistically different from those with normal adrenal function in levels of serum creatinine or bilirubin, MELD score, or presence of cirrhosis related complications. Significant differences were seen in mean international normalized ratio and serum sodium. Patients with a sodium level < 135 mEq/L had a higher rate (31.25%) of adrenal insufficiency.

**Conclusion:**

Adrenal dysfunction was identified in a population of stable nonhospitalized cirrhotic patients. Our results suggest a possible role for adrenal dysfunction as a contributing factor in hyponatremia in cirrhosis independent of other known factors of neurohormonal activation secondary to systemic vasodilation.

## 1. Introduction

Liver cirrhosis has increasingly become a common cause of mortality and disease worldwide [1]. There are a wide variety of complications resulting from extrahepatic organ malfunction in the setting of liver cirrhosis. Hemodynamic derangements, frequently observed in more advanced stages of liver disease, are among the most challenging to treat. Circulatory failure resulting from decreased peripheral vascular resistance and systemic vasodilation, low mean arterial pressure, and poor response to vasopressors could be terminal events in advanced cirrhosis. These homodynamic alterations in cirrhosis share many features with adrenal dysfunction in critically ill patients referred to as “critical illness-related corticosteroid insufficiency” [2, 3].

Adrenal insufficiency has been revealed to be a common complication among critically ill patients with liver disease [4–6]. However, even among patients with stable liver cirrhosis, adrenal dysfunction (if investigated) may not be a rare finding [7, 8]. Proposed as “hepatoadrenal syndrome” [4], adrenal insufficiency in the setting of liver disease is shown to be associated with a high mortality rate [9, 10]. Cirrhotic patients with relative adrenal insufficiency are demonstrated to be at greater risks of circulatory derangement, severe sepsis, renal function impairment, and even hepatorenal syndrome type 1 [9].

The exact mechanism of adrenal insufficiency in cirrhosis is not well understood; however, several mechanisms are suggested. One of these proposed mechanisms is the role of cholesterol and its synthesis impairment in liver disease. Cholesterol is an essential precursor for cortisol synthesis by adrenal gland. In cirrhosis the metabolism of lipoproteins is impaired and low levels of total cholesterol, high-density lipoprotein, and low-density lipoprotein are frequent findings [11, 12]. Low substrate level can lead to the decrease in the production of cortisol by the adrenal in cirrhosis especially in stress conditions. As another mechanism, there could be a possible role for negative feedback of proinflammatory cytokines (which are shown to have increased circulatory level in cirrhosis) on hypothalamic-pituitary-adrenal axis [13]. Adrenal hemorrhage in the setting of impaired coagulation in liver disease also has been implicated as a rare cause of adrenal insufficiency [13].

Prevalence of adrenal insufficiency in studies of patients with liver disease is found to be very variable, perhaps due to the heterogenicity of index cases in addition to different definitional criteria and methods of diagnosis [14, 15]. Most of these studies were on critically ill or decompensated cirrhotic patients, most during hospitalization. The present study is among the few that have evaluated adrenal function and associated factors in stable cirrhotic patients in an outpatient setting.

## 2. Material and Methods

The study included patients with liver cirrhosis referred to the outpatient liver clinics of Shiraz University of Medical Sciences. A diagnosis of cirrhosis was made based on liver histology, clinical manifestations and complications of cirrhosis, imaging findings, and/or a combination of these. Patients with a diagnosis of autoimmune hepatitis and those who had received steroid therapy for any reason during the last 6 months before study were excluded from the study. Critical illness, sepsis, and active infection were among other exclusion criteria. These included recent hospitalization for any critical illness or history of documented bacterial infection or receiving oral or parenteral antibiotic therapy within last 30 days before enrolment. The study was approved by the ethics committee of Shiraz University of Medical Sciences with reference number of EC-91-6369. Every patient gave informed consent prior to their participation in this study.

A total number of 105 patients were included in the study. Demographic data, physical findings, and the results of routine laboratory tests were recorded. Severity of liver disease was graded using model for end-stage liver disease (MELD). Patients were also classified at study entry according to their Child-Turcotte-Pugh (CTP) score and class using laboratory and clinical data. Patients were also classified into compensated cirrhosis and decompensated cirrhosis groups. Decompensation was defined as presence of any of the following conditions: history of ascites, variceal bleeding, or encephalopathy.

For evaluation of adrenal function, adrenocorticotropic hormone (ACTH) stimulation testing was performed on every subject in the Endocrinology and Metabolism Research Center lab. The test was done in the morning between 7:00 and 8:00 am after a 12 hr fast. Baseline cortisol level testing was performed on 2 cc blood drawn from each patient by venipuncture and stored in a colt tube. Then 250 *μ*g of synthetic short acting ACTH was injected intravenously, and, after 60 minutes, another blood sample (2 cc) was taken for cortisol level measurement. Radio immunoassay (RIA) kit was used to measure the level of cortisol in collected plasma samples.

As our patients were not in critically ill condition, adrenal insufficiency was defined as a post ACTH stimulating serum cortisol level of less than 20 mcg/dl (550 nmol/L) [13–15].

Test results were divided into two groups: group 1 included patients with normal adrenal function and group 2 those with adrenal insufficiency.

Statistical analyses were conducted using Statistical Package for Social Sciences (SPSS, Chicago, IL, USA) release 16.0 for Windows. Nonparametric Mann–Whitney test was applied for comparing means of the two groups. Chi square test was used to determine whether the frequency of variables such as presence of ascites, history of gastrointestinal bleeding, encephalopathy, and spontaneous bacterial peritonitis are statistically significant across the two groups. Univariate analysis was used to define the factors associated with adrenal insufficiency. Multiple logistic regression models with backward elimination method were also employed to investigate the correlation of variables with adrenal insufficiency. Covariates with *p* values less than 0.05 were considered to have significant correlation. A receiver operator characteristic (ROC) curve was used to determine a cut-off value for serum sodium level to predict adrenal insufficiency. Area under the curve (AUC) more than 0.6 was considered acceptable. All *p* values less than 0.05 were considered significant.

## 3. Results

The study subjects were consisted of 105 cirrhotic patients, 74 males, ages 15 to 68 years, with a mean age of 40.5 ± 12.4 (±SD) years. Of the patients, 14 were Child class A, 50 were Child class B, and 41 were Child C. The mean MELD score was 18.19 ± 5.46 SD in all cases. Eighty-three patients (79%) had MELD scores of 15 or higher and were considered to be listed for liver transplant. Of all studied patients, 94 were classified as decompensated cirrhosis and only 11 were in the compensated cirrhosis group.

According to defined criteria, adrenal insufficiency was detected in 16 patients (15.23%).

Patients with normal adrenal function (group 1) were not different from group 2 in mean age and sex distribution. The two groups were not different with respect to levels of albumin, creatinine, total bilirubin, or CTP score and MELD score. However, group 2 patients had statistically significantly higher mean INR and lower mean serum sodium levels (see Table 1). Other factors such as presence and severity of ascites, encephalopathy, and history of spontaneous bacterial peritonitis or gastrointestinal bleeding were also investigated for the two groups (Table 1).

Patients with compensated cirrhosis were not statistically different from decompensated ones in the prevalence of adrenal insufficiency (18.2% versus 14.9%  *p* = 0.673), although the number of compensated patients was too small for separate analysis.

**Figure 1:**
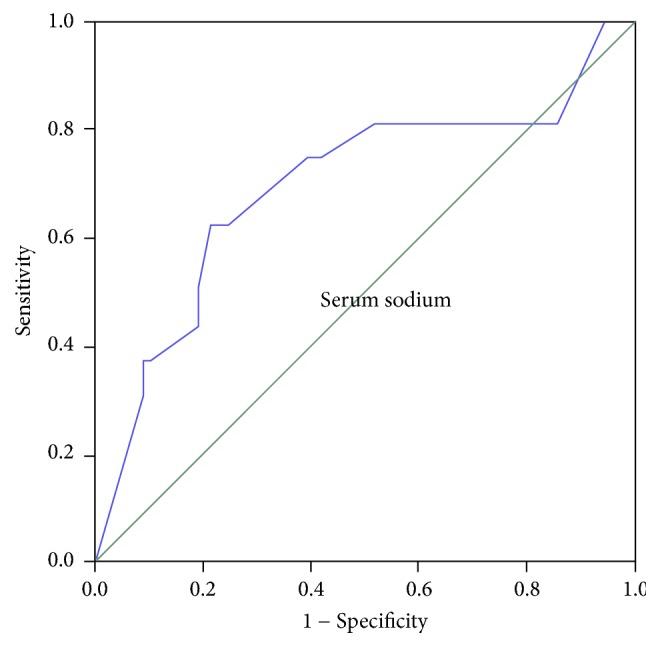
Receiver operator characteristic (ROC) curve for serum sodium in cirrhotic patients tested for adrenal insufficiency.

According to univariate analysis, while serum albumin, total bilirubin, CTP score, and MELD score were not associated with adrenal insufficiency, serum sodium and INR level showed significant associations (*p* < 0.05). The lack of association with adrenal insufficiency was also true for presence of ascites and history of gastrointestinal bleeding, encephalopathy, and spontaneous bacterial peritonitis. Using ROC curve analysis, the cut-off level of serum sodium with optimal sensitivity and specificity for association with adrenal insufficiency was determined to be 133 mEq/L (sensitivity: 0.625, specificity: 0.787) with AUC of 0.697. (Figure 1). To provide a better interpretation of the effect of serum sodium level, subjects were subsequently categorized to two groups, those below and those above 133 mEq/L. As expected, the group with lower sodium showed significant correlation with adrenal insufficiency (OR, 6.41 [95% CI, 1.980–19.047], *p* = 0.02). It was shown that, for every 1 mEq/L sodium level less than 133, there was a 6 times chance of adrenal insufficiency in our patients. However, categorization of the serum sodium level did not change our findings principally. Accordingly, in the multiple logistic model including both INR and serum sodium as categorical variable using cut-off of 133 mEq/L, INR was removed (*p* = 0.001).

The two variables, serum sodium level and serum potassium level, showed significant correlation but in reverse direction (*p* = 0.001) where the two variables moved in opposite directions. Regarding this correlation and in order to prevent colinearity, potassium was not entered in the multiple logistic model while sodium was present.

**Figure 2:**
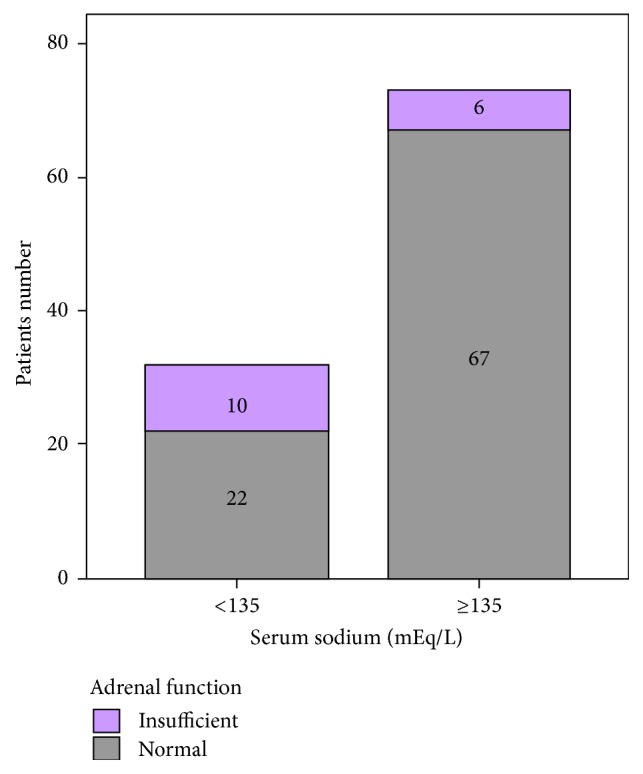
Adrenal function in cirrhotic patients with hyponatremia versus nonhyponatremic ones.

Regarding these results we decided to evaluate the rate of adrenal insufficiency in hyponatremic patients in our study group and compare them with those who did not have hyponatremia. Serum sodium level less than 135 mEq/L was regarded as hyponatremia [16]. Thirty-two of 105 patients (30.5%) were hyponatremic versus 73 (69.5%) with serum sodium levels ≥ 135 mEq/L. Patient with hyponatremia had significantly higher prevalence of adrenal insufficiency (31.25% versus 8.22% and *p* = 0.003) (Figure 2).

## 4. Discussion

The present study revealed that impaired adrenal function was present in 15.23% of nonhospitalized patients with liver cirrhosis, a population where adrenal function is not routinely tested. The considerable prevalence of this complication among critically ill patients with liver cirrhosis has been established by previous studies. In an intensive care unit (ICU) based study, the rate of adrenal insufficiency was reported to be 66% among patients with decompensated cirrhosis and 33% among those with acute liver failure [4]. In another study, again in an ICU setting, a rate of 29.9% was reported in cirrhotic patients with gastroesophageal variceal bleeding [3]. Rates as high as 68% and 51% were reported in cirrhosis with severe sepsis or septic shock, by others [5, 6]. The rate for adrenal insufficiency in our patients is lower than for those in studies of hospitalized cirrhotics. However, our study is one of only a few that was done in stable cirrhotic patients [15]. Adrenal insufficiency is reported to be a more common complication in cirrhosis with variceal bleeding compared with stable hospitalized cirrhotic patients [8]. Even in studies conducted on patients with stable cirrhosis, variable rates have been reported for this complication. Different methods of testing, inhomogeneity of aimed patient population regarding the etiology of liver disease and presence of complications, and status of hospitalization are among the factors contributing to this difference. In contrast with most of the similar studies, all of the patients we included in this study were selected from outpatient clinics and were not hospitalized. Patients were from both sexes and included patients with a wide variety of severity of liver disease (MELD scores: 6 to more than 40) and many etiologies, except for autoimmune hepatitis where steroid use would have been a confounding factor.

In this study the indices of severity of liver disease, MELD and CPT score, although higher in the group with adrenal insufficiency, did not significantly differ from patients with normal adrenal function. A similar finding was reported in the study by Acevedo et al. who did not find any correlation between disease severity and relative adrenal insufficiency in their hospitalized patients with acute decompensation of cirrhosis but who were not critically ill [9]. However, significantly higher levels of INR in our patients with adrenal insufficiency may be indicative of more impaired liver function. High INR is an index of impaired synthetic function of liver and is one of the three variable components in the MELD formula, and the one with the highest coefficient [17, 18].

The major finding of our study is the association of hyponatremia with adrenal dysfunction in our patient population. In logistic models, serum sodium level (whether continuous or categorical) was shown to have the highest relation with adrenal insufficiency among other evaluated factors. This finding was also similar to the finding reported by Acevedo et al. [9]. Hyponatremia is a known complication in advanced cirrhosis and harbors a grave prognosis [19–21]. The mechanism of development of hyponatremia in liver cirrhosis is similar to what happens in heart failure [22]. Arterial underfilling resulting from systemic vasodilation in cirrhosis plays a key role in compensatory activation of neurohormonal system [23]. Nonosmotic release of arginine vasopressin (AVP), activation of sympathetic nervous system, and rennin-angiotensin are among these compensatory mechanisms [22, 24]. The result of action of these mechanisms is sodium and water retention and dilutional hyponatremia.

Glucocorticoid deficiency, on the other hand, may be associated with hyponatremia. With glucocorticoid deficiency, the hypotonic suppression of the osmostat for AVP release is impaired. The results of persistent secretion of AVP are clinical features similar to the syndrome of inappropriate secretion of antidiuretic hormone (SIADH). Although the exact mechanism for this phenomenon is not well defined, the lack of tonically inhibitory effect of glucocorticoids on AVP which responds to glucocorticoid administration may be contributing to this [22, 25].

Our finding of high rate of hyponatremia in patients with cirrhosis and adrenal insufficiency and its reverse (high rate of adrenal insufficiency in cirrhosis with hyponatremia) might be attributed to adrenal dysfunction itself rather than just a symptom of more advanced circulatory dysfunction in patients. It was suggested that adrenal dysfunction may play a role in the development of cardiomyopathy and hepatorenal syndrome in cirrhosis [26]. High plasma renin activity and norepinephrine concentration and also a high probability of hepatorenal syndrome type 1 have been shown in cirrhotic patients with relative adrenal insufficiency [9]. According to the results of ours and similar studies, it can be concluded that adrenal dysfunction could be considered as one of the factors contributing to pathogenesis of hemodynamic alteration in cirrhosis.

## 5. Conclusion

In summary, our study showed that adrenal insufficiency is not a rare complication in cirrhosis, even in nonhospitalized patients. The relation of adrenal insufficiency with low serum sodium level points to the possible role for adrenal dysfunction in hemodynamic instability observed in cirrhosis. The effect of medical treatment of adrenal insufficiency on the management of hyponatremia, circulatory failure, and prevention of resultant complications in cirrhosis such as hepatorenal syndrome could be the subject of future studies.

## Figures and Tables

**Table 1:** Demographic data and clinical characteristics of all patients, patients with normal adrenal function (group 1) and adrenal insufficiency (group 2).

	All patients	Group 1 (Normal Adrenal Function)	Group 2 (Adrenal Insufficiency)	*p* values for differences between group 1 and 2
Age	40.5 ± 12.4	40.53 ± 12.97	40.38 ± 11.84	0.963
Male sex (%)	74 (70.48%)	60 (67.4%)	14 (87.5%)	0.141
MELD score	18.19 ± 5.46	17.88 ± 5.38	19.94 ± 5.73	0.188
CTP score	8.70 ± 1.67	8.61 ± 1.69	9.25 ± 1.53	0.193
Total bilirubin (mg/dl)	3.49 ± 3.04	3.55 ± 3.22	3.15 ± 1.7	0.64
Albumin (mg/dl)	3.4 ± 0.61	3.45 ± 0.59	3.13 ± 0.68	0.095
Creatinine (mg/dl)	1.11 ± 0.65	1.1 ± 0.69	1.17 ± 0.25	0.099
INR	1.97 ± 0.94	1.88 ± 0.81	2.52 ± 1.35	0.032
Sodium (mEq/L)	137.06 ± 7.43	137.88 ± 7.16	132.5 ± 7.53	0.012
Hyponatremia (%)	32 (30.5%)	10 (62.5%)	22 (27.8%)	0.006
Potassium (mEq/L)	4.2 ± 0.54	4.15 ± 0.51	4.5 ± 0.59	0.045
Encephalopathy (%)	79 (75.2%)	70 (78.7%)	9 (56.3%)	0.067
Ascites (%)	34 (32.4%)	32 (36%)	2 (12.5%)	0.065
History of GIB (%)	55 (52.4%)	45 (50.6%)	10 (62.5%)	0.379
History of SBP (%)	46 (43.8%)	41 (46.1%)	5 (31.25%)	0.271

Data are presented as mean ± standard deviation; MELD: model for end stage liver disease; CTP: Child-Turcotte-Pugh; INR: international normalized ratio; GIB: gastrointestinal bleeding; SBP: spontaneous bacterial peritonitis.

## Abbreviations

ACTH: Adrenocorticotropic hormone
AUC: Area under the curve
AVP: Arginine vasopressin
CTP: Child-Turcotte-Pugh
ICU: Intensive care unit
INR: International normalized ratio
MELD: Model for end-stage liver disease
RIA: Radio immunoassay
ROC: Receiver operator characteristic
SIADH: Inappropriate secretion of antidiuretic hormone.
